# Social–Environmental Burden Is Associated with Increased Colorectal Cancer Mortality in Metropolitan Detroit

**DOI:** 10.1158/2767-9764.CRC-24-0503

**Published:** 2025-04-28

**Authors:** Natalie G. Snider-Hoy, R. Blake Buchalter, Theresa A. Hastert, Gregory Dyson, Carina Gronlund, Julie J. Ruterbusch, Ann G. Schwartz, Elena M. Stoffel, Laura S. Rozek, Kristen S. Purrington

**Affiliations:** 1 Department of Oncology, Wayne State University School of Medicine, Detroit, Michigan.; 2 Population Science and Disparities Research Program, Barbara Ann Karmanos Cancer Institute, Detroit, Michigan.; 3 Department of Quantitative Health Sciences, Lerner Research Institute, Cleveland Clinic, Cleveland, Ohio.; 4 Social Environment and Health Program, Survey Research Center, University of Michigan Institute for Social Research, Ann Arbor, Michigan.; 5 Division of Gastroenterology, Department of Internal Medicine, University of Michigan Health System, Ann Arbor, Michigan.; 6 Department of Oncology, Georgetown University School of Medicine, Washington, District of Columbia.

## Abstract

Neighborhood quality affects both socioeconomic factors and exposure to carcinogenic environmental pollutants, but the impacts of these factors on racial disparities in colorectal cancer mortality are not well described. We used the Centers for Disease Control and Prevention Environmental Justice Index social vulnerability module, environmental burden module (EBM), and the combined social–environmental score (SER) to assess relationships with colorectal cancer mortality by race and age in the Metropolitan Detroit Cancer Surveillance System. Among 13,505 patients with colorectal cancer [9,727 non-Hispanic White (NHW) and 3,778 non-Hispanic Black (NHB)], EBM quartile 4 versus 1 was more strongly associated with mortality among NHB early-onset (EO) cases than NHW EO cases [NHB: HR = 1.98, 95% confidence interval (CI), 1.20–3.26; NHW: HR = 1.40, 95% CI, 0.88–2.25]. SER quartile 4 versus 1 was more strongly associated with colorectal cancer mortality in EO (NHB: HR = 1.76, 95% CI, 0.93–3.31; NHW: HR = 1.53, 95% CI, 0.79–2.96) compared with later-onset cases (NHB: HR = 1.15, 95% CI, 0.87–1.52; NHW: HR = 1.39, 95% CI, 1.17–1.65) regardless of race. These associations in EO cases were strongest in colon tumors versus rectal tumors (EO EBM: colon HR = 2.08, 95% CI, 1.24–3.48 vs. rectum HR = 1.03, 95% CI, 0.64–1.66; EO SER: colon HR = 2.57, 95% CI, 1.38–4.79 vs. rectum HR = 0.84, 95% CI, 0.48–1.45). These results suggest the combination of socio-environmental burdens contributes to age and racial disparities in colorectal cancer mortality in metropolitan Detroit.

**Significance:**

Understanding the role of environmental justice in cancer survivorship could influence policy decisions, aiding intervention practices.

## Introduction

Colorectal cancer ranks third in estimated new cancer deaths in the United States in 2024 (1). Whereas colorectal cancer mortality has declined over the last several decades (2), racial disparities in colorectal cancer outcomes remain. Non-Hispanic Black (NHB) patients with colorectal cancer have a 34% greater risk of mortality compared with all other racial groups (1). Unlike the majority of colorectal cancer in the United States, cases diagnosed before the age of 50 [i.e., early-onset (EO) colorectal cancer; ref. 3] have been on the rise since the 1990s (4), and EO colorectal cancer mortality rates are greater among NHB patients compared with other racial groups (5). Understanding the causes of these disparities, especially given the rise of EO colorectal cancer and its disparities by race, is the first step to reducing their burden.

The social construct of race (6) is associated with worse health outcomes among minoritized populations, reflecting differences in socioeconomic conditions and lived experiences (7). Social determinants of health (SDOH) are the health-driving factors that make up an individual’s social, economic, and physical environments (8). Differences in SDOH stem from systemic injustices and racist policies (9), and adverse SDOHs are more prevalent among minoritized populations (10) in the United States, leading to poorer health outcomes (8, 9). Specifically, SDOHs are influenced by both societal norms that perpetuate racial disparities, known as systemic racism, as well as societal barriers such as laws and policies that continue to reinforce racial inequities, known as structural racism (11).

Neighborhood quality is among the most well-studied SDOH in cancer health disparities, often evaluated using area-level socioeconomic metrics (12–14). Inequities in neighborhood quality are known to be heavily related to historical redlining, a key example of structural racism in which the Federal Housing Administration refused to insure mortgages in predominantly Black neighborhoods. This policy and accompanying practices have disenfranchised Black households and households of color by limiting homeownership and encouraging White families to purchase homes in expanding suburbs, which eventually led to widespread divestment in Black neighborhoods in cities (15). Historical redlining practices in metropolitan Detroit have resulted in long-lasting geographic segregation and racial inequity in neighborhood quality within Detroit and its suburbs (16, 17), and we and others have shown that lower neighborhood quality heightens racial disparities in colorectal cancer (18–20).

In addition to increased social vulnerability related to housing quality, employment, and education opportunities, racial minorities often experience higher pollution exposure (21–23). It has been shown in many studies that redlined neighborhoods have higher concentrations of air, soil, and water pollution compared with non-redlined neighborhoods (24–27), metropolitan Detroit included (28, 29), as redlined neighborhoods were often purposefully chosen for the development of highways and industrial facilities (30, 31). Environmental pollution is related to many poor health outcomes in diseases such as cardiovascular disease, respiratory disease (32–34), and cancer (35–41). Though lifestyle factors like diet, tobacco use, and alcohol use are important in colorectal cancer development and progression (42), it is important to note that ambient air pollution such as particulate matter and nitrogen oxides, heavy metals found in polluted soil such as lead, arsenic, and cadmium, and polluted surface water have all been tied to an increased risk of developing colorectal cancer or colorectal cancer mortality in many recent studies (43–53). These contaminants are thought to increase inflammation in the colon, induce oxidative damage, and alter epigenetic marks, all important biological mechanisms in the development and progression of colorectal cancer (54–56). Therefore, it is possible that increased exposure to environmental pollutants as a result of historical redlining may contribute to racial disparities in colorectal cancer mortality in metropolitan Detroit.

Nevertheless, few studies investigate disparities in colorectal cancer survival related to environmental pollution by race, and even fewer account for the concurrent effects of socioeconomic status (SES) related to environmentally vulnerable neighborhoods. The effects of socioeconomic and environmental vulnerability on cancer mortality are expected to accumulate over multiple years. The combined risk from exposure to multiple biological, chemical, physical, and psychosocial stressors has been utilized by the U.S. Environmental Protection Agency (EPA) to provide a broader and more holistic approach to understanding the combined effects of multiple exposures on outcomes (57), such as socioeconomic and environmental disparities on colorectal cancer survival. Our study assesses the role of environmental burden, social vulnerability, and their combined burden on disparities of colorectal cancer mortality in metropolitan Detroit by both age and race by utilizing a comprehensive tool called the Environmental Justice Index (EJI). This tool has been utilized in several other peer-reviewed studies to assess the relationships between social and environmental burden and adverse pregnancy outcomes (58), cardiovascular disease risk (59), and childhood asthma (60), deeming the EJI an appropriate tool to utilize in our own study.

## Materials and Methods

### Identification of colorectal cancer cases in metropolitan Detroit

The Metropolitan Detroit Cancer Surveillance System (MDCSS) registry catchment area contains the three metropolitan counties of Detroit: Oakland, Macomb, and Wayne (county containing the city of Detroit). All three counties were included because they encompass our entire catchment area, and they contain urban, suburban, and rural neighborhood environments, which are useful to compare exposure status outside of the more segregated areas within Detroit, which is the largest city in the area. The non-Detroit neighborhoods are used as a referent population. We identified all incident invasive colorectal cancers diagnosed in Wayne, Oakland, and Macomb counties in Michigan between January 1, 2010, and December 31, 2019, using the MDCSS registry. Individuals with less than 1 month of follow-up were excluded, amounting to 14,159 participants. Due to a low sample size of 347 across two other ethnic minority groups, we removed participants who were not non-Hispanic White (NHW) or NHB based on self-reported race and ethnicity, leading to a final sample size of 13,505. The MDCSS is a founding member of the Surveillance, Epidemiology, and End Results Program and has been continuously collecting population-based cancer data since 1973. This study was classified as non-human subjects research by the Wayne State University Institutional Review Board.

### Clinical and demographic variables

Treatment, clinical, and outcomes data were obtained through the MDCSS registry. These data include age at diagnosis, sex, grade, Surveillance, Epidemiology, and End Results summary stage, insurance status, surgery status, radiation status, vital status, cause of death, and date of last contact. The MDCSS conducts active follow-up for the purpose of determining vital status and survival time after diagnosis, but changes in address are not recorded in such a way that allows the construction of postdiagnosis residential histories. These covariates were selected by *a priori* evidence for being related to the outcome of mortality and to increase precision of estimates, as well as their availability within our registry. Census tract at diagnosis was obtained using the Federal Information Processing Standards convention to match the EJI variables of each patient.

### EJI

The EJI was created by the Centers for Disease Control to rank the cumulative impacts of environmental justice on health for every U.S. census tract (61). The EJI utilizes 36 environmental, social, and health factors, which are grouped into three overarching modules. In our analyses, we used the social vulnerability module (SVM), the environmental burden module (EBM), and a combination of the SVM and EBM called the social environmental score (SER). A detailed list of the variables included in all three modules is included in Table 1. Variables within the SVM accounted for factors such as racial/ethnic minority status, SES, household characteristics, and housing types. All SVM variables were taken from the U.S. Census Bureau American Community Survey from data years 2015 to 2019. Variables within the EBM accounted for factors such as air pollution (U.S. EPA Air Quality System; data years: 2014–2016 and 2014), potentially hazardous and toxic sites (U.S. EPA Facility Registry Service, U.S. Mine Safety and Health Administration Mine Data Retrieval System; data year: 2021), built environment (TomTom MultiNet Enterprise Dataset, U.S. Census Bureau American Community Survey, U.S. EPA National Walkability Index; data years: 2020, 2015–2019, and 2021), transportation infrastructure (TomTom MultiNet Enterprise Dataset, data year: 2020), and water pollution (U.S. EPA Watershed Index Online, data year: 2019). Each module is converted to a national percentile ranking. The SER adds the SVM and EBM percentiles together and then creates a new percentile variable; therefore, the modules are weighted equally. The SER module was created for researching health outcomes (62). To utilize these data in our models, we turned the continuous percentile variables into quartile levels based among the distribution of all three counties, providing an effective method for comparison. The EJI 2022 edition was utilized for these models as the times of measurement of the predictors within the modules are relevant to our time of diagnosis (62). Most area-level metrics do not change substantially year to year; thus, variables measured within and around the years of diagnosis will be appropriate to apply to our participants. Additionally, the 2022 edition is the first available version of this unique and useful publicly available dataset.

**Table 1:** EJI predictors with variable information and data years

Themes	Individual variables	Data year
Social vulnerability–variable information		
Racial/ethnic minority status	Minority status	2015–2019
SES	Poverty No high school diploma Unemployment Housing tenure Housing burdened lower-income households Lack of health insurance Lack of broadband access	2015–2019
Household characteristics	Age 65 and older Age 17 and younger Civilian with a disability Speaks English “less than well”	2015–2019
Housing type	Group quarters Mobile homes	2015–2019
Environmental burden–variable information		
Air pollution	Ozone PM_2.5_ Diesel particulate matter Air toxicity cancer risk	2014–2016 2014–2016 2014 2014
Potentially hazardous and toxic sites	National priority list sites Toxic release inventory sites Treatment, storage, and disposal sites Risk management plan sites Coal mines Lead mines	2021
Built environment	Recreational parks Houses built before 1980 Walkability	2020 2015–2019 2021
Transportation infrastructure	High-volume roads Railways Airports	2020
Water pollution	Impaired surface water	2019

### Statistical analysis

All data were analyzed using RStudio statistical software (https://cran.r-project.org/; RRID: SCR_000432). Descriptive statistics were used to characterize the data. Associations between categorical variables were evaluated using the χ^2^ test except for age, which was compared using the Student *t* test. Cox proportional hazards (PH) regression was used to estimate associations between overall survival and cancer-specific survival utilizing the “coxph” function in the *survival* R package (RRID: SCR_021137; ref. 63). HRs and 95% confidence intervals (CI) for each EJI variable were reported. Variables in Cox PH models were evaluated for violation of PH assumptions utilizing the “cox.zph” function. Models accounted for covariates, including age at diagnosis, sex, receival of radiotherapy, receival of surgery, tumor stage, and insurance status. Our motivation for using the EJI was that it lends itself to the separate and combined evaluation of the social and environmental subcomponents through the SVM, EBM, and SER variables. Due to substantial collinearity between these variables, we did not adjust SVM models for EBM and *vice versa*. As such, evaluations of the relative contributions of these variables were done by visually comparing effect estimates of the SVM and EBM models individually as well as in comparison with the combined SER model. All statistical tests were two-sided, with a *P* value of <0.05 considered statistically significant. We use the *P* value as a general rule to come to comparable conclusions while also considering effect sizes and CIs to analyze patterns, trends, and associations (64).

### Mapping figures

Maps of EJI variables were created using GeoDa software (RRID: SCR_018559; ref. 65).

### Data availability

The data generated in this study are not publicly available due to patient privacy. Deidentified data may be made available based on requests submitted via the MDCSS website (https://www.karmanos.org/karmanos/epidemiology-research-core) and based on approval of the MDCSS leadership.

## Results

### Patient demographics

A total of 13,505 invasive primary colorectal cancer cases (9,272 NHB and 3,778 NHW) with at least 1 month of follow-up were identified (Table 2). NHW patients had a higher proportion of males compared with NHB patients (51.4% vs. 48.8%, *P* = 0.020). Average age at diagnosis was lower among NHB patients than NHW patients (62.7 vs. 66.0, *P* < 0.0001), and NHB patients had a higher proportion of EO disease compared with NHW patients (14.3% vs. 12.2%, *P* = 0.0010). NHW patients had higher proportions of regional tumors (39.1% vs. 32.3%, *P* < 0.0001) and rectal tumors (31.2% vs. 27.8%, *P* < 0.0001). Tumor grade showed no clear patterns of association by race, although differences in grade distribution by race were statistically significant (*P* < 0.0001).

**Table 2:** Patient demographics

		NHW		NHB		
		*n* = 9,727		*n* = 3,778		
		*n*	%	*n*	%	*P* value
Patient and tumor characteristics						
Sex	Male	5,005	51.45%	1,844	48.81%	0.02
	Female	4,722	48.55%	1,934	51.19%	
Age at diagnosis	Mean (SD)	66.0 (14.4)		62.7 (13.2)		
EO	Under 50	1,191	12.24%	540	14.29%	0.001
	50 or older	8,536	87.76%	3,238	85.71%	
Stage at diagnosis	Localized	3,497	35.95%	1,453	38.46%	<0.0001
	Regional	3,798	39.05%	1,240	32.82%	
	Distant	2,169	22.30%	970	25.67%	
	Unstaged	263	2.70%	115	3.04%	
Grade	I	977	10.04%	418	11.06%	<0.0001
	II	5,384	55.35%	2,049	54.24%	
	III	1,210	12.44%	347	9.18%	
	IV	198	2.04%	50	1.32%	
	Unknown	1,958	20.13%	914	24.19%	
Anatomic site	Colon	6,689	68.77%	2,729	72.23%	<0.0001
	Rectum	3,038	31.23%	1,049	27.77%	
Insurance type	Private	3,682	37.85%	1,402	37.11%	0.43
	Other	6,045	62.15%	2,376	62.89%	
Treatment characteristics						
Radiation	Radiation	1,651	16.97%	391	10.35%	<0.0001
	None/unknown	8,076	83.03%	3,387	89.65%	
Surgery	Received surgery	1,582	16.26%	829	21.94%	<0.0001
	Did not receive surgery/unknown	8,145	83.73%	2,949	78.06%	
Vital status	Deceased	5,005	51.45%	2,078	55.00%	0.0002
	Alive	4,722	48.55%	1,700	45.00%	

**Figure 1:**
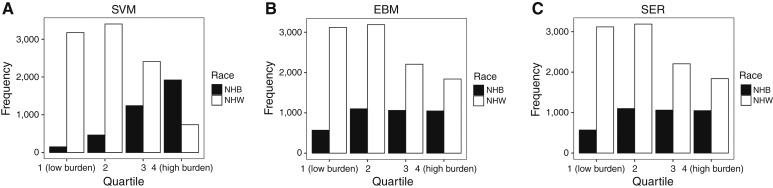
Racial distributions of patients by EJI quartiles (shown as counts). **A,** SVM, (**B**) EBM, and (**C**) SER.

NHB patients were less likely to receive radiotherapy than NHW patients (10.4% vs. 17.0%, *P* < 0.0001) but more likely to receive surgery (21.9% vs. 16.3%, *P* < 0.0001). Interestingly, there was no difference in the use of private insurance by race (*P* = 0.43). NHB patients were also more likely to live in areas of higher social vulnerability [SVM quartile 4 (Q4) 50.9% vs. 7.6%, *P* < 0.0001], environmental burden (EBM Q4 27.7% vs. 18.9%, *P* < 0.0001), and combined social–environmental burden (SER; Q4 43.4% vs. 10.6%, *P* < 0.0001; Fig. 1A–C).

### Geospatial distribution of EJI variables

**Figure 2:**
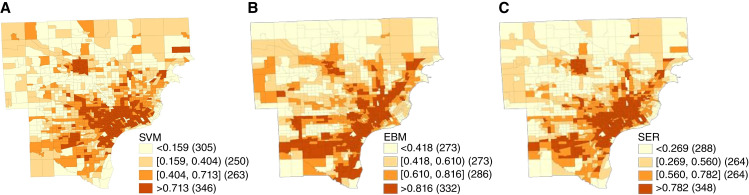
Geographic distribution of EJI variables in metropolitan Detroit at the census tract level for (**A**) SVM raw scores categorized by quartile, (**B**) EBM raw scores categorized by quartile, and (**C**) combined SER raw scores categorized by quartile. For each variable, Q1 is depicted in light yellow, Q2 in light orange, Q3 in medium orange, and Q4 in dark orange. Raw score values corresponding to each quartile are shown in the legends. Census tracts without sufficient data to calculate scores are shown in black.

The EJI variables at the percentile level were mapped based on their quartile breakdowns in the final analyses. Among all three variables, we note the city of Detroit always experienced the highest rates of burden (darkest orange). The SVM map (Fig. 2A) depicts the highest burden (Q4: >0.816), most concentrated toward the city. The EBM map (Fig. 2B) shows more widespread elevated burden, especially along highway routes (interstates 94 and 96), as well as the downriver region south of the city. The SER map (Fig. 2C) shows a combination of the two variables, as expected due to the calculation method of this variable.

### Associations with overall and colorectal cancer–specific mortality by race

We first analyzed the effects of the SVM, EBM, and SER on overall mortality by race and age of diagnosis (Table 3). For ease of presentation, we discuss the HRs for mortality for the highest quartile (Q4) compared with the lowest quartile (Q1). All three EJI variables consistently increased the risk of mortality across strata, although not all associations were statistically significant. SVM Q4 versus Q1 increased the risk of mortality with similar effects by race (NHW: HR = 1.36, 95% CI, 1.21–1.51; NHB: HR = 1.30, 95% CI, 1.01–1.67). The EBM was also associated with increased mortality across strata, although the effects were slightly weaker in magnitude (NHW: HR = 1.22, 95% CI, 1.12–1.32, NHB: HR = 1.08, 95% CI, 0.94–1.24) and not statistically significant among NHB patients. The combined SER variable increased the risk of mortality with similar effect sizes compared with the effects of the SVM alone, in which the SER increased risk by 37% (95% CI, 24%–50%) among NHW patients and by 31% (95% CI, 6%–62%) among NHB patients.

**Table 3:** Associations between EJI variables and overall and cancer-specific mortality among all patients stratified by race

		NHW			NHB		
		HR	95% CI	*P* value	HR	95% CI	*P* value
Overall survival							
SVM	Q2 vs. Q1	1.14	(1.06–1.22)	0.00024	0.99	(0.74–1.31)	0.93
	Q3 vs. Q1	1.28	(1.19–1.38)	<0.0001	1.19	(0.92–1.54)	0.20
	Q4 vs. Q1	1.36	(1.21–1.51)	<0.0001	1.30	(1.01–1.67)	0.044
	*P* for trend			<0.0001			<0.0001
EBM	Q2 vs. Q1	1.04	(0.97–1.13)	0.25	0.94	(0.81–1.08)	0.38
	Q3 vs. Q1	1.15	(1.07–1.25)	0.00021	1.05	(0.91–1.21)	0.48
	Q4 vs. Q1	1.22	(1.12–1.32)	<0.0001	1.08	(0.94–1.24)	0.29
	*P* for trend			<0.0001			0.050
SER	Q2 vs. Q1	1.07	(0.99–1.14)	0.077	0.95	(0.74–1.20)	0.65
	Q3 vs. Q1	1.26	(1.17–1.36)	<0.0001	1.24	(1.00–1.54)	0.050
	Q4 vs. Q1	1.37	(1.24–1.50)	<0.0001	1.31	(1.06–1.62)	0.012
	*P* for trend			<0.0001			<0.0001
		*n* = 9,720		Events = 5,002	*n* = 3,772		Events = 2,073
Cancer-specific survival							
SVM	Q2 vs. Q1	1.10	(0.98–1.25)	0.11	1.44	(0.82–2.54)	0.20
	Q3 vs. Q1	1.23	(1.08–1.40)	0.002	1.60	(0.94–2.71)	0.083
	Q4 vs. Q1	1.34	(1.11–1.63)	0.003	1.74	(1.03–2.94)	0.037
	*P* for trend			0.00015			0.026
EBM	Q2 vs. Q1	1.02	(0.90–1.16)	0.77	0.97	(0.73–1.29)	0.83
	Q3 vs. Q1	1.17	(1.03–1.34)	0.020	1.38	(1.03–1.84)	0.031
	Q4 vs. Q1	1.17	(1.02–1.35)	0.027	1.31	(0.99–1.74)	0.057
	*P* for trend			0.0057			0.038
SER	Q2 vs. Q1	1.03	(0.91–1.16)	0.65	0.98	(0.63–1.51)	0.92
	Q3 vs. Q1	1.20	(1.05–1.37)	0.0073	1.29	(0.87–1.90)	0.21
	Q4 vs. Q1	1.40	(1.19–1.66)	<0.0001	1.52	(1.04–2.23)	0.031
	*P* for trend			<0.0001			0.00058
		*n* = 7,462		Events = 2,744	*n* = 2,915		Events = 1,216

We next determined the effects of EJI variables on cancer-specific mortality by race, which were similar to the effects observed for overall mortality (Table 3). The SVM was associated with about a 74% increase in risk of colorectal cancer–specific mortality among NHB patients, with a smaller effect among NHW patients (NHW: HR = 1.34, 95% CI, 1.11–1.63; NHB: HR = 1.74, 95% CI, 1.03–2.94). The EBM was related to higher risk of colorectal cancer–specific mortality among NHB patients, though not statistically significant at the 0.05 level. The SER increased the risk of mortality among both groups, again greater in NHB patients. There was no evidence for effect modification of EJI variables by race for either overall or cancer-specific mortality.

### EJI variable associations by race and age of onset

Given that EJI variable effects were very similar for overall compared with colorectal cancer–specific mortality, all subsequent subset analyses are presented only for colorectal cancer–specific mortality. Stratified by race and age of onset (Table 4), the SVM was most substantially associated with colorectal cancer–specific survival among EO NHW patients (HR = 2.12, 95% CI, 0.97–4.61) compared with all other groups. Interestingly, the EBM was associated with a nearly 2-fold increase in colorectal cancer–specific mortality only among EO NHB patients (HR = 1.98, 95% CI, 1.20–3.26). The combined SER variable was greatest among EO NHB patients and greater than that of EO NHW patients, whereas the opposite was true among late-onset (LO) patients, only at a smaller magnitude. Whereas the SER effect among EO NHB patients seems to be driven primarily by the EBM, its effect among EO NHW patients seems to reflect the combined effects of the SVM and EBM even though neither variable was statistically significant. The SER was also significantly associated with a 39% increase in colorectal cancer–specific mortality for LO NHW patients (HR = 1.39, 95% CI, 1.17–1.66) but was not associated with mortality among LO NHB patients.

**Table 4:** Associations between EJI variables and cancer-specific mortality stratified by race and age of onset

		NHW			NHB		
		HR	95% CI	*P* value	HR	95% CI	*P* value
EO (<50)							
SVM	Q2 vs. Q1	1.09	(0.73–1.61)	0.68	0.70	(0.35–1.41)	0.32
	Q3 vs. Q1	1.19	(0.77–1.84)	0.42	0.87	(0.47–1.59)	0.64
	Q4 vs. Q1	2.12	(0.97–4.61)	0.059	1.10	(0.61–2.00)	0.75
	*P* for trend			0.11			0.18
EBM	Q2 vs. Q1	0.90	(0.59–1.37)	0.63	2.07	(1.26–3.40)	0.0042
	Q3 vs. Q1	0.88	(0.56–1.39)	0.59	1.78	(1.09–2.92)	0.022
	Q4 vs. Q1	1.40	(0.88–2.25)	0.16	1.98	(1.20–3.26)	0.0071
	*P* for trend			0.30			0.030
SER	Q2 vs. Q1	1.22	(0.84–1.78)	0.30	1.25	(0.62–2.51)	0.54
	Q3 vs. Q1	1.35	(0.86–2.12)	0.19	1.40	(0.50–2.62)	0.29
	Q4 vs. Q1	1.53	(0.79–2.96)	0.21	1.76	(0.93–3.31)	0.082
	*P* for trend			0.10			0.039
		*n* = 1,320		Events = 351	*n* = 579		Events = 186
LO (50+)							
SVM	Q2 vs. Q1	1.10	(0.97–1.25)	0.12	1.12	(0.75–1.69)	0.58
	Q3 vs. Q1	1.22	(1.07–1.41)	0.0029	1.17	(0.80–1.72)	0.41
	Q4 vs. Q1	1.31	(1.07–1.60)	0.0091	1.24	(0.85–1.80)	0.26
	*P* for trend			0.00051			0.15
EBM	Q2 vs. Q1	1.03	(0.90–1.19)	0.63	0.92	(0.75–1.13)	0.43
	Q3 vs. Q1	1.20	(1.05–1.38)	0.0097	1.08	(0.88–1.32)	0.45
	Q4 vs. Q1	1.16	(1.00–1.34)	0.057	1.09	(0.89–1.33)	0.39
	*P* for trend			0.010			0.091
SER	Q2 vs. Q1	1.01	(0.89–1.14)	0.90	0.91	(0.66–1.25)	0.57
	Q3 vs. Q1	1.19	(1.03–1.37)	0.018	1.07	(0.81–1.43)	0.62
	Q4 vs. Q1	1.39	(1.17–1.65)	0.0002	1.15	(0.87–1.52)	0.31
	*P* for trend			<0.0001			0.031
		*n* = 6,158		Events = 2,392	*n* = 2,336		Events = 1,030

### EJI variable associations by anatomic site, race, and age of onset

We further explored the EJI variable associations described above separately for colon versus rectal tumors. Among NHW patients, associations between all EJI variables and colorectal cancer–specific survival were largely consistent between colon and rectal tumors (Table 5) and consistent with effects seen among all tumor sites combined (Table 3). The SVM and SER were associated with 27% to 56% increases in colorectal cancer–specific mortality risk for both colon (SVM: HR = 1.27, 95% CI, 1.01–1.61; SER: HR = 1.37, 95% CI, 1.12–1.68) and rectal (SVM: HR = 1.56, 95% CI, 1.06–2.31; SER: HR = 1.39, 95% CI, 0.97–2.00) cancers in NHW patients. This pattern was also observed among NHB patients stratified by site, although with less statistical significance.

**Table 5:** Associations between EJI variables and cancer-specific mortality stratified by anatomic site and race

		Colon			Rectum		
		HR	95% CI	*P* value	HR	95% CI	*P* value
NHW							
SVM	Q2 vs. Q1	0.97	(0.85–1.12)	0.71	1.39	(1.05–1.84)	0.020
	Q3 vs. Q1	1.11	(0.95–1.29)	0.19	1.40	(1.05–1.88)	0.024
	Q4 vs. Q1	1.27	(1.01–1.61)	0.043	1.56	(1.06–2.31)	0.25
	*P* for trend			0.034			0.011
EBM	Q2 vs. Q1	1.00	(0.85–1.16)	0.96	1.08	(0.81–1.44)	0.61
	Q3 vs. Q1	1.14	(0.97–1.33)	0.11	1.01	(0.75–1.36)	0.90
	Q4 vs. Q1	1.14	(0.97–1.35)	0.12	1.14	(0.83–1.50)	0.42
	*P* for trend			0.049			0.55
SER	Q2 vs. Q1	0.97	(0.84–1.11)	0.65	1.24	(0.95–1.61)	0.11
	Q3 vs. Q1	1.11	(0.94–1.30)	0.21	1.23	(0.92–1.66)	0.17
	Q4 vs. Q1	1.37	(1.12–1.68)	0.0020	1.39	(0.97–2.00)	0.070
	*P* for trend			0.0037			0.57
		*n* = 5,077		Events = 1,975	*n* = 2,385		Events = 769
NHB							
SVM	Q2 vs. Q1	0.98	(0.67–1.45)	0.93	0.98	(0.47–2.06)	0.97
	Q3 vs. Q1	1.12	(0.78–1.60)	0.54	1.37	(0.70–2.67)	0.35
	Q4 vs. Q1	1.22	(0.86–1.73)	0.26	1.35	(0.70–2.61)	0.36
	*P* for trend			0.030			0.15
EBM	Q2 vs. Q1	0.94	(0.76–1.17)	0.60	1.26	(0.85–1.88)	0.25
	Q3 vs. Q1	1.20	(0.97–1.49)	0.095	1.27	(0.84–1.91)	0.26
	Q4 vs. Q1	1.22	(0.98–1.50)	0.070	1.29	(0.88–1.91)	0.20
	*P* for trend			0.0052			0.29
SER	Q2 vs. Q1	0.89	(0.64–1.23)	0.47	1.16	(0.61–2.21)	0.65
	Q3 vs. Q1	1.09	(0.81–1.46)	0.58	1.47	(0.84–2.58)	0.18
	Q4 vs. Q1	1.27	(0.95–1.70)	0.10	1.51	(0.86–2.65)	0.15
	*P* for trend			0.00081			0.087
		*n* = 2,084		Events = 945	*n* = 830		Events = 271

Considering stratification by anatomic site among EO cancers (Table 6), associations between EJI variables and colorectal cancer–specific mortality were observed only among colon tumors, with stronger effects than those observed in the overall analysis for NHW or NHB patients (Table 2). The SVM was associated with a 1.80 increase in colorectal cancer–specific mortality among EO patients with colon tumors (95% CI, 0.95–3.40). The EBM (HR = 2.04, 95% CI, 1.20–3.45) and SER (HR = 2.54, 95% CI, 1.46–4.43) were both associated with more than 2-fold increases in colorectal cancer–specific mortality risk among colon tumors. However, the results differed among LO patients. In the LO group, only the SVM (colon: HR = 1.29, 95% CI, 1.08–1.55; rectal: HR = 1.53, 95% CI, 1.13–2.07) and SER (colon: HR = 1.36, 95% CI, 1.15–1.61; rectal: HR = 1.40, 95% CI, 1.04–1.88) variables were associated with increased risk of colorectal cancer–specific mortality among LO cancers. Though these results were very interesting, we were unable to further stratify these analyses by race due to the small sample size.

**Table 6:** Associations between EJI variables and cancer-specific mortality stratified by anatomic site and age of onset

		Colon			Rectum		
		HR	95% CI	*P* value	HR	95% CI	*P* value
EO							
SVM	Q2 vs. Q1	0.95	(0.59–1.55)	0.85	0.80	(0.54–1.20)	0.28
	Q3 vs. Q1	1.32	(0.79–2.20)	0.29	0.78	(0.50–1.22)	0.28
	Q4 vs. Q1	1.80	(0.95–3.40)	0.072	0.85	(0.48–1.51)	0.57
	*P* for trend			0.060			0.37
EBM	Q2 vs. Q1	1.22	(0.76–1.95)	0.42	1.38	(0.92–2.07)	0.12
	Q3 vs. Q1	1.26	(0.75–2.11)	0.38	1.11	(0.73–1.69)	0.63
	Q4 vs. Q1	2.08	(1.24–3.48)	0.0053	1.03	(0.64–1.66)	0.89
	*P* for trend			0.0069			0.99
SER	Q2 vs. Q1	1.41	(0.71–1.83)	0.59	1.14	(0.77–1.68)	0.52
	Q3 vs. Q1	1.89	(1.13–3.17)	0.015	0.87	(0.55–1.36)	0.53
	Q4 vs. Q1	2.57	(1.38–4.79)	0.003	0.84	(0.48–1.45)	0.53
	*P* for trend			0.0013			0.40
		*n* = 1,158		Events = 362	*n* = 724		Events = 175
LO							
SVM	Q2 vs. Q1	0.99	(0.86–1.14)	0.85	1.53	(1.18–1.99)	0.00014
	Q3 vs. Q1	1.11	(0.95–1.28)	0.18	1.45	(1.11–1.89)	0.068
	Q4 vs. Q1	1.29	(1.08–1.55)	0.0063	1.53	(1.12–2.10)	0.0079
	*P* for trend			0.0065			0.014
EBM	Q2 vs. Q1	0.95	(0.82–1.10)	0.48	1.06	(0.82–1.36)	0.66
	Q3 vs. Q1	1.16	(1.00–1.34)	0.045	1.08	(0.83–1.39)	0.58
	Q4 vs. Q1	1.11	(0.96–1.29)	0.16	1.19	(0.91–1.55)	0.21
	*P* for trend			0.030			0.23
SER	Q2 vs. Q1	0.93	(0.81–1.07)	0.32	1.27	(0.98–1.63)	0.067
	Q3 vs. Q1	1.09	(0.94–1.26)	0.27	1.43	(1.10–1.87)	0.0078
	Q4 vs. Q1	1.36	(1.15–1.61)	0.00029	1.40	(1.04–1.88)	0.0025
	*P* for trend			0.00035			0.014
		*n* = 6,003		Events = 2,558	*n* = 2,492		Events = 865

## Discussion

We evaluated socioeconomic, environmental, and combined social–environmental vulnerabilities and colorectal cancer mortality in metropolitan Detroit with respect to age and race. Studies have shown the effect of SES on colorectal cancer mortality is well understood and wide reaching (18, 19, 66, 67), whereas the effect of environmental burden is less consistent. Given that drivers of EO colorectal cancer risk and mortality are not well understood, our findings that the effects of EBM were elevated compared with the effects of SVM in this group are novel to the field. Our findings with respect to the effect of social factors on colorectal cancer mortality are comparable with those presented in the many cancer disparities studies that have previously utilized the Social Vulnerability Index/Module. A nationwide county-level study comparing social vulnerability and cancer mortality found similar risks of mortality of all cancer types between NHB [Q4 vs. Q1 rate ratio (RR) = 1.11 (1.08–1.13)] and NHW patients [Q4 vs. Q1 RR = 1.10 (1.09–1.11); ref. 68]. Colorectal cancer–specific mortality was slightly elevated [Q4 vs. Q1 RR = 1.15 (1.13–1.17)]; however, this study did not further stratify by race. These estimates may be lower than those in our study due to the elevated segregation and localized disadvantage in metropolitan Detroit compared with the entire country. An additional nationwide county-level study found counties with high Social Vulnerability Index/Module scores to have increased risk of colorectal cancer mortality [Q5 vs. Q1 RR = 1.23 (1.15–1.31); ref. 69]. Another study based in Texas and California found that increased social vulnerability reduced the odds of a patient receiving surgery for early-stage colon cancer as well as increased mortality (70). They also found that patients above the age of 65 were more sensitive to variation in social vulnerability, which we noticed in Table 4 between our EO and LO patients.

To our knowledge, no currently published studies have specifically utilized the EBM of the EJI to investigate racial disparities in colorectal cancer mortality. Whereas other metrics of environmental exposures have been utilized to study disparities, most are limited to single exposures, such as air quality. Many studies have found relationships between environmental exposures like air pollutants, heavy metals, and contaminated water and an increase in colorectal cancer incidence or mortality (43–53). However, none of these studies reported risk estimates stratified by race or age of onset, a key distinction of our study. Other studies have investigated the role of air pollution on racial disparities in morbidity and mortality in the United States using various endpoints such as lung cancer, stroke, and ischemic heart disease, along with pollutant-attributable deaths, finding that Black and Hispanic individuals are consistently more heavily affected than White individuals (71, 72). To better understand how environmental pollution can affect cancer health disparities, it is essential we investigate the effects on incidence and mortality by both age and race in other racially diverse metropolitan areas.

It is well established that SES and environmental exposures are often intertwined (73), and it can be difficult to evaluate individual effects due to collinearity. The development of the combined SER from the EJI allows us to effectively account for both factors while investigating colorectal cancer disparities, as they are equally weighted in this variable. To our knowledge, no study has utilized a combined social–environmental burden variable to study disparities in colorectal cancer. However, other groups have used the SER variable to study disparities in other health outcomes. A study on pregnancy outcomes in New York found NHB patients had an elevated risk of adverse pregnancy outcomes related to SER status compared with NHW patients [adjusted OR 1.668, (95% CI, 1.581–1.760); ref. 58]. Another study on childhood asthma in Atlanta found that children in areas of high SER had greater asthma exacerbation occurrence, worse asthma control questionnaire scores, and lower forced expiratory volume compared with those who lived in areas of moderate to low SER (60). Implementing this new metric in additional studies across cancer sites and geographic locations has significant potential to advance our understanding of the relative contributions of social and environmental factors in cancer health disparities.

Our study has several strengths. Our large sample size of 13,505 participants from the population-based MDCSS registry provides generalizability and validity to our results. The catchment area of the MDCSS is a racially diverse and segregated area, which makes it an ideal location to study the effects of social–environmental burden on NHB cancer survivorship compared with NHW patients with adequate power. These results can likely be generalized to other racially segregated cities in the United States (New Orleans, Memphis, Chicago, Atlanta, etc.), especially those with elevated rates of environmental pollution, such as Cancer Alley (74–76) in Louisiana. We found our NHB patients typically lived in areas of higher social and environmental burden in Detroit, opposite of the pattern shown for NHW patients, a trend we expected based on existing literature (21–23, 28).

Another strength to our study is the use of the EJI. The EJI was created by the Centers for Disease Control as a call for federal tools to address cumulative impacts of environmental injustice on health (62) by identifying communities facing the worst impacts of environmental burden. Cumulative impact assessment can be utilized as an alternative to traditional risk and exposure assessment. In large retrospective population-based studies, it is difficult to carefully track and match exposure of a single pollutant to a patient over time with high confidence of completeness, accuracy, and resolution of spatial differences (77). By utilizing a combination of quantitative and semi-quantitative information, we can evaluate a comprehensive estimate of risk of colorectal cancer mortality related to social vulnerability, environmental burden, and the potential synergy of social–environmental effects at the quartile level. Again, this study does not point to any specific variable causing disparities in colorectal cancer mortality but rather a high-level measure of impacts coming from multiple known sources over time related to social vulnerability or environmental burden.

Our study was limited by the lack of available individual-level exposure data such as smoking status, chemotherapy treatment, diet, and body mass index. As some of these factors are correlated with socioeconomic disadvantage, it is possible we captured at least some of the effects utilizing insurance status or the SVM. In particular, racial differences in guideline-concordant treatment for colorectal cancer are an important consideration for differences in colorectal cancer mortality, although treatment disparities have been shown to be improving in recent years (78). We also were unable to account for residential movement after diagnosis or workplace exposure, which has the possibility to impact cumulative exposure assessment (79). However, some studies suggest that a patient’s residence at the time of diagnosis is most likely representative of the neighborhood exposures measured here due to the lifetime SES the patient has experienced (80). One longitudinal study in the Midwest found that mobility rates overall and mobility-related exposure misclassification were low across 20 years of follow-up (81). A study of residential mobility and environmental inequality found that mobility due to financial hardship usually results in higher pollution exposures (82), so we would expect that misclassification related to financial hardship in our study would result in overly conservative estimates. Furthermore, the Nashville Health and Stress Study showed that the benefits of upward SES mobility were not only dependent on the lifetime trajectory of SES but were also not as pronounced among Black compared with White individuals (80). Lastly, this study is also limited to only NHB and NHW patients with colorectal cancer due to small sample sizes of other minority populations in our catchment area.

Additionally, the EJI does not contain all potential environmental justice issues a community may face. The EJI also has limitations regarding timing, which relates to the diagnosis time range of our patients. Pollutants such as PM_2.5_ and ozone have been declining over the last decade (83, 84), thus, the measure of these pollutants from 2014 to 2016 utilized in the EJI are a rough estimate of relative exposure levels for our patients diagnosed between 2010 and 2019 and their progression toward mortality. Also, area-level metrics of SES change slowly over time, thus, these metrics are likely accurate. We utilized the first edition of the EJI created in 2022, which uses measurements closest to our patient’s years of diagnosis. Lastly, the environmental indicators used in the EJI do not represent detailed measures of risk or exposure assessment but rather an overall description of environmental burden facing a community. Our study compares the risk of mortality relative to the exposure of social/environmental burden but not specific measures of the concentration of pollutants or exact proximity to hazardous sites. Despite these limitations, the use of cumulative burden to estimate increases in risk of mortality in colorectal cancer is an ideal metric to study disparities in both age and race related to social–environmental burden.

In summary, we found individual and combined social–environmental burden variables increased the risk of colorectal cancer mortality in most analyses. The largest effects were associated with the SER among EO patients, particularly those with colon tumors. Due to the biological implications of environmental pollutants and cancer, our future directions include the molecular profiling of NHW and NHB colorectal cancer patient tumors in metropolitan Detroit to compare to the patient’s social–environmental burden. As EO patients seemed to have an elevated risk of mortality related to environmental exposure, we are particularly interested in the molecular landscapes of these tumors, which will help us better understand the risk factors, behavior, and potential molecular targets associated with EO colorectal cancer.
